# Supplementary material for: Comparison of Human Embryonic Stem Cell-Derived Cardiomyocytes, Cardiovascular Progenitors, and Bone Marrow Mononuclear Cells for Cardiac Repair
Source: Stem Cell Reports. 2015 Oct 22;5(5):753–62. doi: 10.1016/j.stemcr.2015.09.011 (PMC4649260; doi:10.1016/j.stemcr.2015.09.011)
Supplement: Document S1. Figures S1–S3 and Tables S1–S4 [file mmc1.pdf]

Stem Cell Reports

Supplemental Information

Comparison of Human Embryonic Stem Cell-Derived Cardiomyocytes, Cardiovascular Progenitors, and Bone Marrow Mononuclear Cells for Cardiac Repair

Sarah Fernandes, James J.H. Chong, Sharon L. Paige, Mineo Iwata, Beverly Torok-Storb, Gordon Keller, Hans Reinecke, and Charles E. Murry

Supplementary Data

Supplementary Figures Legend

Supplementary Figure S1. Human ESC differentiation protocols. Human ESC derivatives were obtained from a unique batch of cryopreserved undifferentiated ESC. Human ESCs were depleted of MEFs by at least 2 passages on Matrigel coated plate, before cryopreservation. Non-cardiac derivatives were obtained using a monolayer differentiation protocol. Progenitor and definitive cells were issued from the same differentiation protocol: Cardiovascular progenitors and definitive cells were obtained at day 5 and 15 after the beginning of the differentiation respectively. All cells underwent a heat shock procedure the day before being harvested for intracardiac injection. * Sampling for flow cytometry analysis.

Supplementary Figure S2. Evaluation of vascularization using neutron activated microsphere. a Sample preparation**:** After overnight fixation, hearts were sliced in 5 sections. The basal section was discarded, 2 sections were kept for histology and 2 for microsphere analysis (flow). For blood flow assessment infarcted and uninjured segment were individually isolated, whereas peri infarcted area were discarded. RV, right ventricle; LV, left ventricle**. b** Evaluation of the vascularization using microsphere injections in the non infarcted area did not show any significant difference between the 4 groups at one month time point. **c** Representative RECA staining of BM-MNC group showing that capillary staining and the presence of 2 microspheres (red arrows) within a non infarcted area.

Supplementary Figure S3. Immunophenotyping of human grafts in Non-Cardiac recipients. Photomicrograph of the largestt human graft observed in the Non-Cardiac recipients. Adjacent sections have been stained with Hematoxylin Eosin, human Pan Centromeric probe, beta Myosin Heavy Chain and, pan cytokeratin. Immunostainings confirmed that, in the Non cardiac recipient, human grafts were mostly composed by epithelial derivatives, and were depleted of cardiomyocytes.

Supplementary Table S1. Primary antibodies used for cell immunophenotyping before cell transplantation

Antigen	Antibody Type (Clone Name or Catalog #)	Supplier	Titer
Cardiac troponin T	Mouse monoclonal Clone 11-13 (cat # MS-295)	LabVision/ Neomarkers	1:10
CD117/cKit-APC	Clone 104D2 (cat # 313205)	BioLegend	1:5
CD133-PE	Clone 293 C3 (cat # 130-090-8530)	Miltenyi Biotech	1:5
CD13-APC	Clone WM15 (cat # 5574540)	BD Biosciences	1:5
CD146-FITC	Clone P1H12 (cat # 5608460)	BD Biosciences	1:5
CD14-PE	Clone MφP3 (cat # 347497)	BD Biosciences	1:5
CD19-FITC	Clone HIB19 (cat # 560994)	BD Biosciences	1:5
CD235a/Glycopholin A-APC	Clone GA-R2(HIR2) (cat # 551336)	BD Biosciences	1:5
CD33-FITC	Clone HIM3-4 (cat # 561818)	BD Biosciences	1:5
CD34-APC	Clone 581 (cat # 555824)	BD Biosciences	1:5
CD3-PE	Clone SK7 (cat # 347347)	BD Biosciences	1:5
CD41a-APC	Clone HIP8 (cat # 559777)	BD Biosciences	1:5
CD45-PE	Clone HI30 (cat # 555484)	BD Biosciences	1:5
CD4-APC	Clone 11830 (cat # FAB3791A)	R&D Systems	1:5
CD8-APC	Clone 37006 (cat # FAB1509A)	R&D Systems	1:5
CD90/THY1-FITC	Clone F15-42-1 (cat # a b11155)	abcam	1:5
human CD31-PerCP-eFlour710	Mouse monoclonal Clone WM-59 (cat # 46-0319-41)	eBioscience	1:10
PDGFRα-APC	Mouse monoclonal Clone PRa292 (cat # FAB1264)	R & D Systems	1:5
smooth muscle actin	Rabbit polyclonal (cat # ab5694)	Abcam	1:5
VEGFR2/KDR-PE	Mouse monoclonal Clone 89106 (cat # FAB357)	R & D Systems	1:5

Supplementary Table S2. Primary antibodies used for histology analysis.

Antigen	Antibody Type (Clone Name or Catalog #)	Supplier	Titer
**Cadherin**	Mouse monoclonal Clone CH19	Sigma-Aldrich	1:100
**Cardiac troponin T**	Mouse monoclonal Clone 11-13 (cat # MS-295)	LabVision/ Neomarkers	1:400
**CD31 (Human-specific)**	Mouse monoclonal (Clone JC70A)	Dako	1:20
**Nkx2.5**	Horse polyclonal (cat.# AF2444)	R&D Systems	1/400
**Pan-cytokeratins**	Mouse monoclonal Clone AE1/AE3	Dako	1:150
**Reca**	Mouse monoclonal RECA- 1 (cat # ab9774)	abcam	1/15
**β3-tubulin**	Mouse monoclonal Clone SDL.3D10	Sigma-Aldrich	1:2000
**β-myosin heavy chain**	Mouse monoclonal Clone A4.951	ATCC	1:10

Abbreviations: ATCC— American Type Culture Collection

Supplementary Table S3. Characterization of BM-MNC preparations.

Antibody	%
CD117/cKit+	5±1
CD133+	<0.1
CD14+	10±1
CD146+	<0.1
CD19+	11±3
CD3+	24±3
CD33+, CD13+	23±3
CD34+	4±1
CD34+/cKit+	2±1
CD4+	12±2
CD41a+	3±1
CD45+	73±4
CD8+	11±2
CD90/Thy1+	<0.5
Glycophorin A+	11±2

Supplementary Table S4: Histomorphometry values of scar size evaluation

Group	Areas obtained by histomorphometry (arbitrary unit)									
	Section 1		Section 2		Section 3		Section 4			Scar/lv %
	LV	Scar	LV	Scar	LV	Scar	LV	Scar		
hESC-CVP	36.9	0.0	42.6	2.4	24.7	5.7	44.2	5.2		10.1
	27.2	0.0	40.4	11.5	20.4	7.3	41.4	4.0		18.5
	42.6	0.0	41.3	4.4	16.9	0.4				4.4
	63.8	5.0	30.6	3.9	48.5	0.0				6.9
									**Average**	**10.0**
hBM-MNC	32.4	5.5	21.8	4.4	30.8	1.0				13.5
	21.9	0.0	38.8	4.7	18.0	1.3	34.3	1.8		8.1
	29.9	0.0	36.3	4.9	30.1	0.0				4.5
	36.5	6.9	16.7	0.8	37.1	2.5				10.2
	45.4	8.8	18.2	4.6	44.4	0.0				14.9
	46.4	0.0	53.0	2.4	36.5	5.8	41.4	0.0		5.1
	34.8	0.0	49.9	4.3	45.8	10.7				10.6
	47.3	6.2	38.8	5.6	14.0	0.2				9.7
	38.0	0.0	45.3	2.0	39.8	4.8				5.5
	55.9	0.0	56.3	11.7	13.2	3.2				15.1
									**Average**	**9.7**
hESC-CM	33.5	1.5	36.9	6.6	21.8	2.7	36.9	2.5		12.3
	53.7	8.7	31.0	5.3	42.7	3.0				13.5
	38.5	5.2	22.1	2.4	38.8	1.8				9.6
	29.6	0.0	52.1	8.5	27.1	1.7	39.9	0.0		7.5
	59.6	0.0	66.7	13.4	28.6	5.1	68.3	0.0		12.6
	49.8	0.0	35.6	7.1	55.5	6.4	57.2	0.0		10.4
	33.7	0.0	47.7	2.2	23.6	0.3				1.9
									**Average**	**9.7**
Non cardiac	58.3	0.0	66.8	7.1	42.8	9.3				10.8
	70.0	0.0	72.8	8.7	35.5	17.1	83.9	11.0		18.3
	71.1	0.0	62.1	9.6	89.1	3.6	22.2	0.0		19.4
	55.1	0.0	49.6	6.9	27.8	2.9				8.1
									**Average**	**14.1**

Evaluation of scar was performed on picrosirius red stained section from base (section 1) to apex (section 3 or 4). Total left ventricular area (LV), scar area and scar/LV ratios were determined for each section. The scar/LV % was obtained by averaging the value of each section.

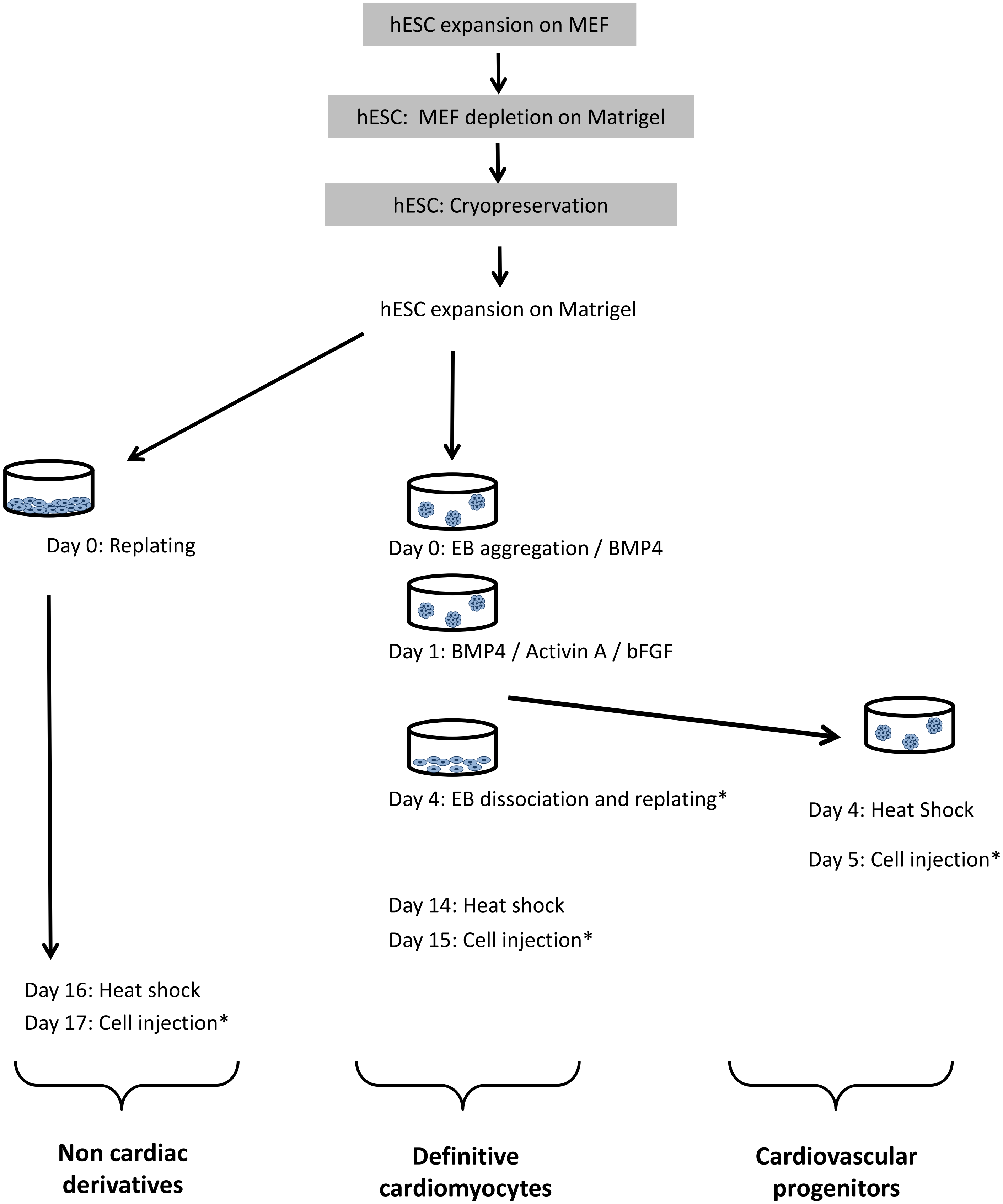

Supplementary figure S1

a

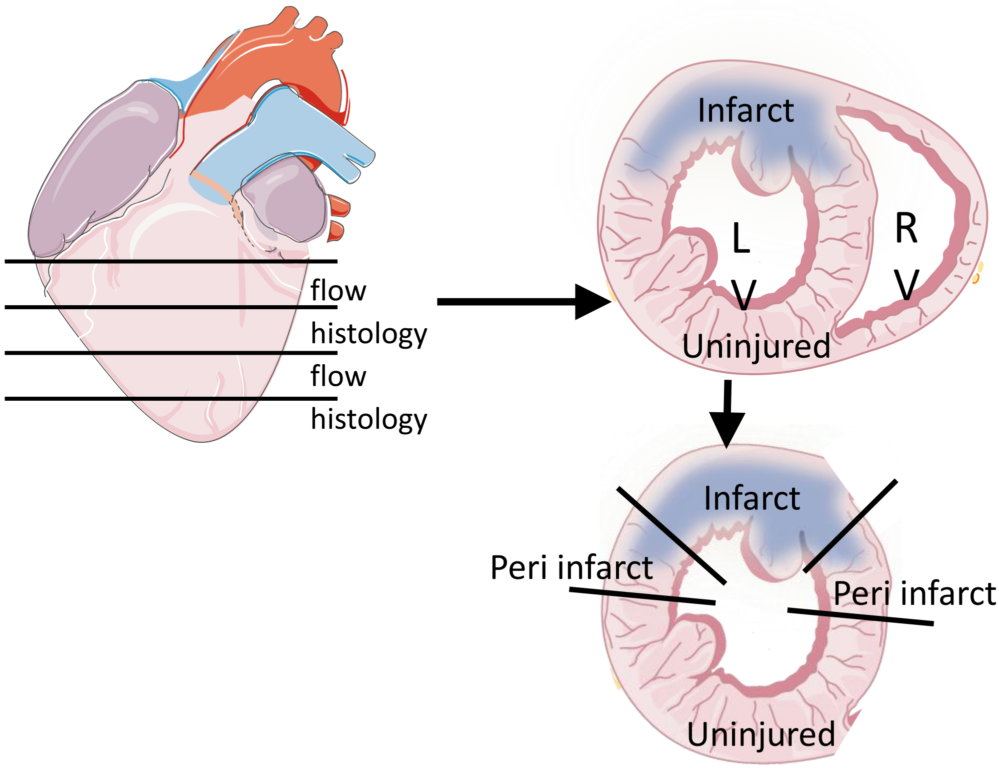

b

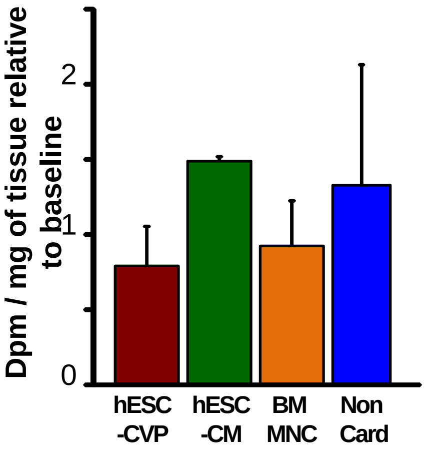

c

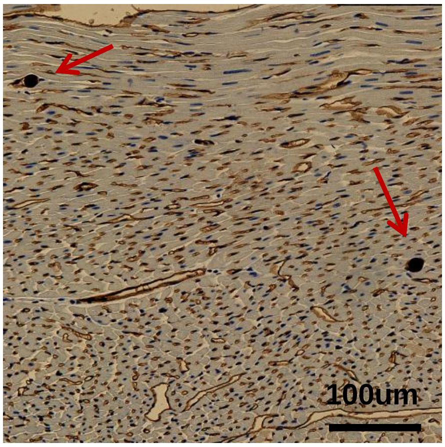

Supplementary Figure S2

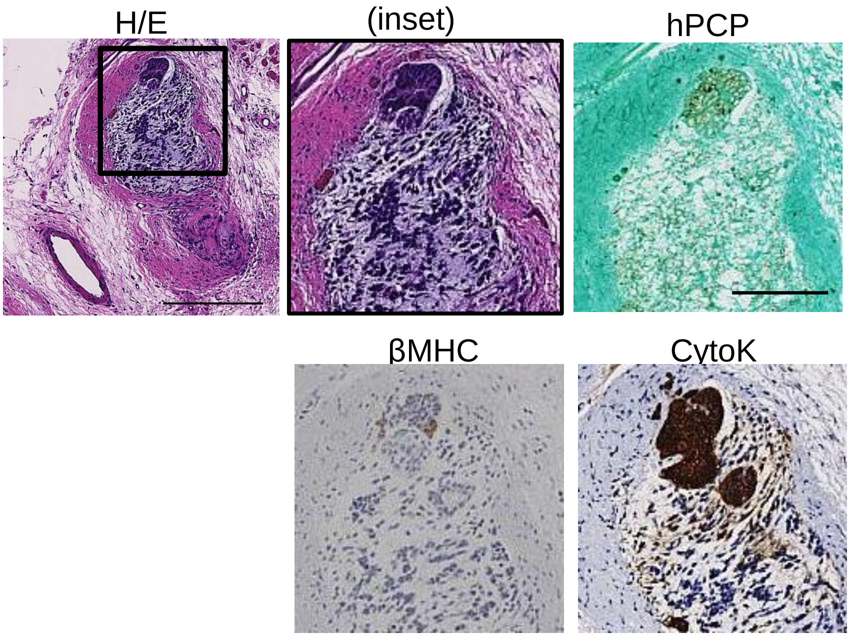

Supplementary Figure S3
