# Supplementary material for: Comparison of Human Embryonic Stem Cell-Derived Cardiomyocytes, Cardiovascular Progenitors, and Bone Marrow Mononuclear Cells for Cardiac Repair
Source: Stem Cell Reports. 2015 Oct 22;5(5):753–62. doi: 10.1016/j.stemcr.2015.09.011 (PMC4649260; doi:10.1016/j.stemcr.2015.09.011)
Supplement: Document S2. Article plus Supplemental Information [file mmc2.pdf]

Stem Cell Reports
Article
OPEN ACCESS

Comparison of Human Embryonic Stem Cell-Derived Cardiomyocytes, Cardiovascular Progenitors, and Bone Marrow Mononuclear Cells for Cardiac Repair

Sarah Fernandes,[1,2,3,10] James J.H. Chong,[1,2,3,4,5,10] Sharon L. Paige,[1,2,3] Mineo Iwata,[8] Beverly Torok-Storb,[8] Gordon Keller,[9] Hans Reinecke,[1,2,3] and Charles E. Murry[1,2,3,6,7,*]
[1]Center for Cardiovascular Biology, University of Washington, Seattle, WA 98109, USA
[2]Institute for Stem Cell and Regenerative Medicine, University of Washington, Seattle, WA 98109, USA
[3]Department of Pathology, University of Washington, Seattle, WA 98109, USA
[4]School of Medicine, University of Sydney, Sydney, NSW 2006, Australia
[5]Westmead Millennium Institute for Medical Research, University of Sydney, Sydney, NSW 2145, Australia
[6]Department of Bioengineering, University of Washington, Seattle, WA 98109, USA
[7]Department of Medicine/Cardiology, University of Washington, Seattle, WA 98109, USA
[8]Fred Hutchinson Cancer Research Center, Seattle, WA 98109, USA
[9]McEwen Centre for Regenerative Medicine, Ontario Cancer Institute, Toronto, ON M5G 2M9, Canada
[10]Co-first author
*Correspondence: murry@uw.edu
http://dx.doi.org/10.1016/j.stemcr.2015.09.011
This is an open access article under the CC BY license (http://creativecommons.org/licenses/by/4.0/).

SUMMARY

Cardiomyocytes derived from human embryonic stem cells (hESC-CMs) can improve the contractility of injured hearts. We hypothesized that mesodermal cardiovascular progenitors (hESC-CVPs), capable of generating vascular cells in addition to cardiomyocytes, would provide superior repair by contributing to multiple components of myocardium. We performed a head-to-head comparison of hESC-CMs and hESC-CVPs and compared these with the most commonly used clinical cell type, human bone marrow mononuclear cells (hBM-MNCs). In a nude rat model of myocardial infarction, hESC-CMs and hESC-CVPs generated comparable grafts. Both similarly improved systolic function and ventricular dilation. Furthermore, only rare human vessels formed from hESC-CVPs. hBM-MNCs attenuated ventricular dilation and enhanced host vascularization without engrafting long-term or improving contractility. Thus, hESC-CMs and CVPs show similar efficacy for cardiac repair, and both are more efficient than hBM-MNCs. However, hESC-CVPs do not form larger grafts or more significant numbers of human vessels in the infarcted heart.

INTRODUCTION

Cell-based cardiac repair is an active research area in both preclinical settings and in clinical trials. Because they are easily accessible, have a favorable safety profile, and have shown efficacy in preclinical studies, autologous bone marrow mononuclear cells (hBM-MNCs) have been the most frequent cell source used in clinical trials. However, these clinical trials have shown discrepant results with some studies demonstrating improved cardiac function and clinical symptoms, whereas others have demonstrated no such improvements (Chong, 2012). In addition, the mechanism of action for hBM-MNC-induced cardiac efficacy remains elusive. It is now accepted that transplanted hBM-MNCs cannot create sufficient amounts of new cardiac muscle for significant contractile force generation. A more likely hypothesis is that their beneficial effect is related to paracrine actions and induction of neoangiogenesis (Dai et al., 2013; Hansson et al., 2009; Kocher et al., 2001; van der Bogt et al., 2008).

Recently, the beneficial effect of cardiomyocytes derived from human embryonic stem cells (hESC-CMs) has been demonstrated in various preclinical models of cardiac injury (Caspi et al., 2007; Chong et al., 2014; Laflamme et al., 2007; Leor et al., 2007; Shiba et al., 2012; van Laake et al., 2008). These studies show that hESC-CMs can engraft and remuscularize the myocardium and preserve the contractile function of the heart when injected shortly after myocardial infarction. Furthermore recent studies have demonstrated that hESC-CM grafts in the injured hearts of guinea pigs and macaques form electromechanical junctions with host cardiomyocytes and contract synchronously with the host heart (Chong et al., 2014; Shiba et al., 2012). However, while hESC-CM treatment can halt the deterioration of cardiac function, they have failed to improve already diminished cardiac function (Fernandes et al., 2010), perhaps because the grafts have only repopulated a small amount of the infarct. Thus, there is clearly room for improvement.

Yang et al. (2008) described a novel population of human tripotent cardiovascular progenitor cells that can be derived from hESCs (hESC-CVPs). This population, identified on the basis of their KDR (VEGFR2)/PDGFRα expression, represents a promising source for heart repair, as these cardiovascular progenitors have a restricted capacity to differentiate into cardiomyocytes, smooth muscle cells,

and vascular endothelium. This specific cell population could, in principle, not only remuscularize the damaged myocardium improving its contractility, but also promote the revascularization of the injured area.

Thus, different cellular sources for cardiac repair remain of considerable interest to the field. However, there is a lack of studies directly comparing different cell types in the same animal model. In the present study, we aimed to determine the fate of three promising cellular sources for cardiac repair—hBM-MNCs, hESC-CVPs, and definitive beating hESC-derived cardiomyocytes (hESC-CMs)—after transplantation into the infarcted rat heart. Furthermore, we assessed their impact on host cardiac remodeling and cardiac function.

RESULTS

Cardiovascular progenitor (hESC-CVP; day 5 of differentiation) and definitive cardiomyocyte (hESC-CM; beating cells at approximately day 15 of differentiation) preparations were obtained by directing differentiation of H7 hESCs toward the cardiovascular lineage. Briefly, cells were allowed to form embryoid bodies in the presence of defined serum-free medium as previously described (Yang et al., 2008). Mesoderm induction was accomplished using bone morphogenetic protein 4 (BMP4), activin A, and basic fibroblast growth factor (BFGF) (Figure S1). On day 5 of differentiation (at the time of the injection procedure), hESC-CVP preparations contained 74% ± 4% tripotential cardiovascular progenitor (from 57% to 92%, identified by flow cytometry based on expression of KDR and PDGFRα; Figure 1B) (Yang et al., 2008). Over time in culture, these mesodermal progenitors gave rise to a cell population that contained predominantly cardiomyocytes (70% ± 11%; from 54% to 91%; cTNT by flow cytometry at day 14) with a small percentage of endothelial cells (1.6% ± 0.1%) and smooth muscle cells (6.5% ± 3.5%), as assessed by flow cytometry for human $CD31^{+}$ and $SMA^{+}/cTNT^{-}$ cells, respectively (Figure 1C).

Fresh hBM-MNC preparations were harvested from the posterior iliac crests of healthy donors. Flow cytometry performed on each preparation at the time of injection (Table S3) demonstrated that 73% ± 4% expressed the common leukocyte antigen CD45, 11% ± 2% expressed the erythroid marker glycophorin A, and 5% ± 1% expressed the progenitor marker c-KIT. As expected, the hBM-MNCs were negative for cardiomyocyte markers (Figure 1D) and contained a low percentage of endothelial cells (1.1% ± 0.4%; $CD31^{+}$). Colony-forming assays in methylcellulose confirmed viability and normal functional properties of the bone marrow product (Figure 1F). These features demonstrate that our hBM-MNC preparations were healthy and representative of normal unfractionated bone marrow.

To determine the physiologic consequences of cell transplantation, we compared the functional outcome following transplantation of these cells to that observed with control hearts receiving non-cardiac hESC neuroectodermal derivatives (a cell population that has been shown not to affect heart function or remodeling after myocardial infarction; Fernandes et al., 2010; Laflamme et al., 2007; Shiba et al., 2012). Non-cardiac derivatives of hESCs (Non-Cardio) were obtained as previously described, using a monolayer derivation protocol without exogenous growth factors (Chong et al., 2014; Fernandes et al., 2010; Laflamme et al., 2007) performed in parallel of the hESC-CVP/hESC-CM differentiation protocol (Figure S1). Those preparations contained virtually no cardiomyocytes (0.3% ± 0.2% by flow cytometry) or endothelial cells (0.1% ± 0.1%) (Figure 1E).

The impact of transplanting these four cell populations was assessed in an athymic rat model of myocardial infarction, induced by 60 min of ischemia followed by reperfusion (Figure 1A). Myocardial infarction was confirmed by echocardiography on the day of cell transplantation (4 days after the ischemia-reperfusion procedure). To ensure comparability among groups, we excluded from the study animals with baseline fractional shortening >40% or that subsequently demonstrated no histologically identifiable infarct. After randomization, rats underwent intra-myocardial injection of either 10×10^{6} cardiovascular progenitor (hESC-CVP; n = 9), definitive cardiomyocytes (hESC-CMs; n = 11), hBM-MNCs (n = 11), or non-cardiac derivatives of hESCs (n = 13). Mortality after cell transplantation is given in Table 1 and ranged from 9% in the hBM-MNC group to 27% in the hESC-CM group.

All physiological studies were conducted and interpreted by investigators blinded to the animal's treatment. Echocardiography (Figure 2A) performed just before transplantation (4 days after ischemia/reperfusion [I/R]) demonstrated that, at this early time point, rats already showed signs of negative remodeling with ventricular dilation and reduced contractile function (Figures 2A and 2C). In a separate cohort of uninjured rats, fractional shortening (FS) averaged 51.0% ± 2.1%, whereas in our infarcted groups, FS ranged from 28.9% ± 1.1% to 31.8% ± 0.8%. Importantly, the pre-transplantation left ventricular dimension (left ventricular end diastolic and end systolic dimension; LVEDD and LVESD, respectively) and FS were similar in all groups (Figure 2C), indicating effective randomization and comparable infarct sizes among the animals.

By 28 days after cell transplantation (32 days post-myocardial infarction), animals that received the non-cardiac cell control population showed significant ventricular dilation (+18%, $p < 0.001$, and +26%, $p < 0.001$, for LVEDD and LVESD, respectively) and decreased fractional shortening (−13%, $p < 0.005$) relative to their baseline

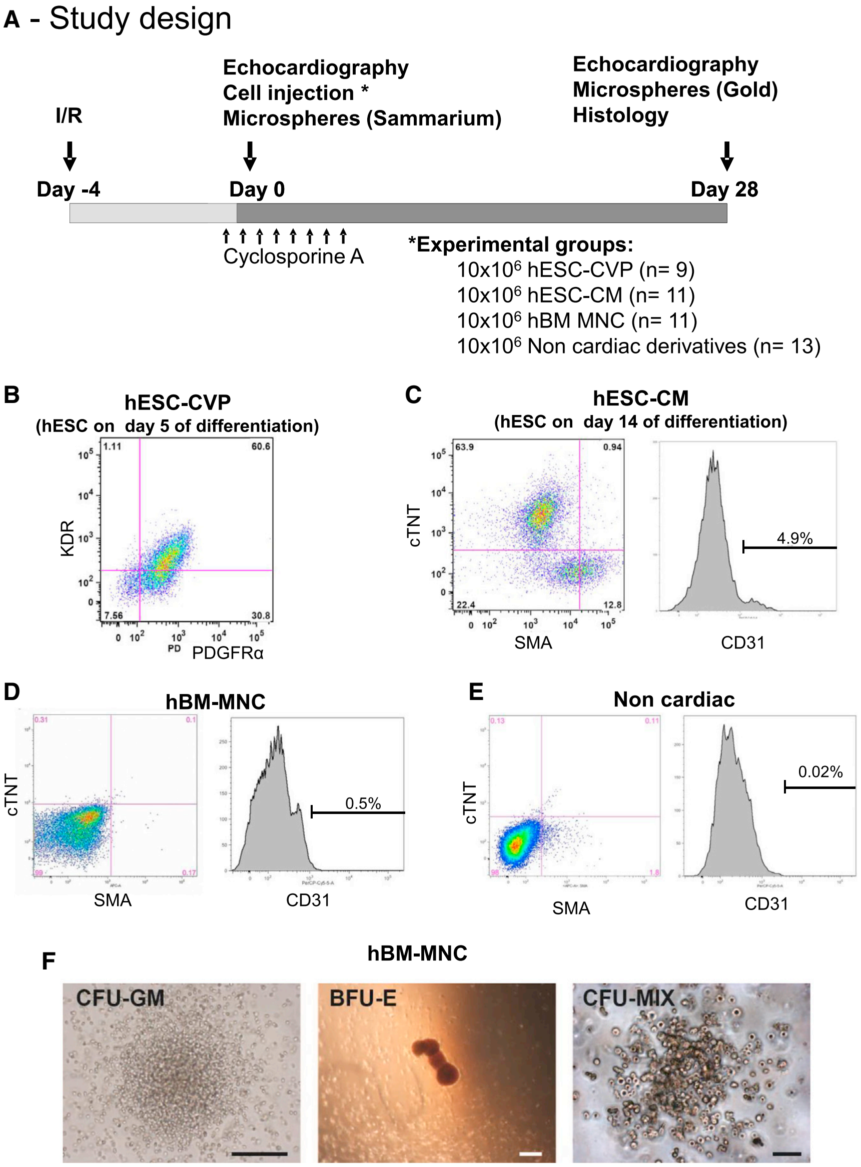

Figure 1. Study Design and Analysis of Cell Preparations

(A) Nude rats were subjected to 60 min ischemia followed by reperfusion. At 4 days after injury, animals underwent a repeat thoracotomy and intra-myocardial injection of either hESC-CVP, hESC-CM, hBM MNC, or non-cardiac derivatives of hESCs in a pro-survival cocktail. To ensure xenograft survival, we treated all animals from days −1 to day +7 post-transplantation with cyclosporine A. Endpoints included echocardiography and measurement of blood flow with microspheres (both performed at days 0 and 28 post-transplantation), and histology (on day +28 post-transplantation).

(B–F) Human cell preparations were characterized by flow cytometry on the day of cell injection. At day 5 after commencement of differentiation, the cardiovascular progenitor population (hESC-CVP preparation) was characterized using the anti-human KDR and anti-human PDGFRα antibody in (B). At day 14 after commencement of differentiation, hESC-CVPs have matured into hESC-CMs, and the percentage of cardiomyocytes, endothelial cell, and smooth muscle cell was evaluated by cTNT/alpha-SMA co-staining and hCD31 immunostaining (C). In BM-MNC and non-cardiac preparations (D and E), the percentage of cardiomyocytes, endothelial cell, and smooth muscle cell was evaluated similarly. Further characterization of BM-MNCs is provided in Table S3. Representative photomicrographs of colonies obtained from a colony-forming unit assay performed on a BM-MNC are shown in (F). BFU-E, red blood cell colonies known as Burst forming unit; CFU-GM, myeloid cell colony; CFU-MIX, mixture of cell types from one multipotent progenitor. Scale bars represent 200 μm for CFU-GM and BFU-E and 50 μm for CFU-MIX.

measurement at 4 days post-infarction (Figures 2B and 2C). Similarly, left ventricular dimension increased in the hBM-MNC group (+10%, $p < 0.005$, and +15%, $p < 0.05$, for LVEDD and LVESD, respectively); however, this group did not demonstrate a significant decline in global systolic function (+0.5% of FS between days 4 and 28; p = NS). In striking contrast, there was no dilation of the left ventricle at end-systole or end-diastole in hESC-CM- and hESC-CVP-injected groups, and the FS improved significantly (+16% and +28% when compared to baseline; $p < 0.001$ and $p < 0.05$, respectively). There were no differences between the hESC-CM and hESC-CVP groups in terms of ventricular dimension or FS, indicating equal potency for restoration of cardiac structure and function.

At 28 days post-transplantation, animals were euthanized, and their hearts were harvested to assess the size, phenotype, and distribution of the graft, as well as the extent of the scar. All animals showed scar formation and thinning of the involved left ventricular free wall at 32 days after ischemia-reperfusion (Figure 3A). The hESC-CVP, hESC-CM, and hBM-MNC groups showed a trend toward smaller scar sizes compared with the non-cardiac group, but this did not achieve statistical significance (10% ± 3.1%, 9.7% ± 1.2%, 9.7% ± 1.5%, and 14.1% ± 2.8% of the left ventricle for the hESC-CVP, hESC-CM,

Table 1. Animal Death after Cell Injection Procedure

	Animals that Received Cell Injection	Acute Death after Cell Injection	Euthanasia after Cell Injection	Animals Excluded Because Quality Control of the Cell Preparation Was Not Met	Animals Included in the Study	Mortality
hESC-CVPs	14	2	0	4	8	2/14 (14%)
hESC-CMs	15	4	0	0	11	4/15 (27%)
BM-MNCs	12	0	1	0	11	1/11 (9%)
Non-cardiac	16	2	1	0	13	3/16 (19%)

hBM-MNC, or Non-Cardiac group, respectively) (Figure 3B). All hESC-CM and hESC-CVP recipient hearts contained islands of human cardiac grafts encapsulated by scar tissue within the left ventricular free wall (Figure 3C). In contrast, surviving human cells were found in only 3 of 11 recipients of non-cardiac derivatives at 28 days post-transplantation. Those human grafts were very small (<0.001% of the left ventricle) and did not contain any cardiomyocytes (negative β-MHC immunostaining) but consisted primarily of epithelial cells (Figure S3). Similar to previous reports (Lolfredo et al., 2011), we did not observe any long-term survival of human-derived cells in hBM-MNC recipients using in situ hybridization to detect human pan-centromeric sequences.

In hESC-CVP and hESC-CM recipients, human grafts were predominantly located within the central regions of the scar. However, small myocardial implants in the peri-infarct (border) zone or within the non-infarcted host tissue were also found. The human origin of the graft was confirmed by in situ hybridization with a human pan-centromeric probe (hPCP; Figures 3C and 3D). Graft size tended to be larger in the hESC-CM recipients versus the hESC-CVP recipients (2.1% ± 0.5% versus 1.2% ± 0.5% of the LV; 17% ± 3% versus 10% ± 6% of the scar; Figure 3E), but this did not achieve statistical significance. Rare microscopic aggregates of epithelial cells were observed in 2 of 9 hESC-CVP and 4 of 11 the hESC-CM recipients, but careful examination of the histology confirmed absence of teratomas (see Figure S3). In both groups, human grafts were composed mainly of cardiomyocytes expressing myosin heavy chain, cardiac troponin cTNT and NKX2.5, and myofibrils with readily identifiable sarcomeres (Figures 3D and 3F). In larger human grafts, we occasionally observed a core composed of non-human-derived cells. Those host-derived cells had a fibroblastic morphology and were negative for endothelial, epithelial, and contractile markers (RECA, pan cytokeratin, and MF20, respectively), and surrounding tissue was rich in collagen (picrosirius red staining; Figure 3C). These void spaces may represent remnants of necrotic cores from cell injection.

To further assess how cell transplantation affected the host tissue, we evaluated the vascularization of the myocardium. Using intraventricular infusion of neutron-activated microspheres at two different time point of the study (at the time of cell injection and at the time of sacrifice), we did not observe evidence of enhanced blood flow in any group at any time. Evaluation of human-derived endothelial cells (by hCD31 immunostaining; Figure 4A) in the hESC-CVP and hESC-CM groups showed rare presence of vessel-like lumens in 1 of 9 and 4 of 11 animals, respectively (fewer than ten cells per animal). However, staining for rat endothelium with RECA confirmed the widespread presence of host-derived vascularization within hESC-CM and hESC-CVP grafts (Figure 4B). We did not observe any vascular density difference between those two groups (Figure 4C). In both cases, vascular lumen density within the human graft was lower than within the surrounding host myocardium (Figure 4B). Similarly, vascular density within the graft-free scar tissue was not significantly different between the four cell-injected groups, suggesting that cell injection or cardiac muscle formation has little effect on the revascularization of the scar (Figure 4D). In contrast, evaluation of the vascular density in myocardial areas remote from the infarcted zone revealed higher lumen density in the hBM-MNC-injected group when compared with the hESC-CVP or the non-cardiac group (Figure 4E). However, we did not observe any significant difference between the hESC-CM, hESC-CVP, and non-cardiac groups, suggesting little effect of human cardiac grafts on angiogenesis.

DISCUSSION

In the present study, we demonstrated that hESC-CVPs can engraft without tumor formation and have a beneficial effect on the negative remodeling of the heart, in an extent similar to fully differentiated cardiomyocytes (hESC-CMs). Then, using a side-by-side comparison, with blinded analysis, we showed that while hBM-MNCs can halt the negative remodeling of the infarcted heart both hESC-CVPs and hESC-CMs had greater beneficial effects than hBM-MNC transplantation on the contractile function of the heart.

Improvement of cardiac function after transplantation of various types of cells has been extensively shown in different animal models and species. However, because of

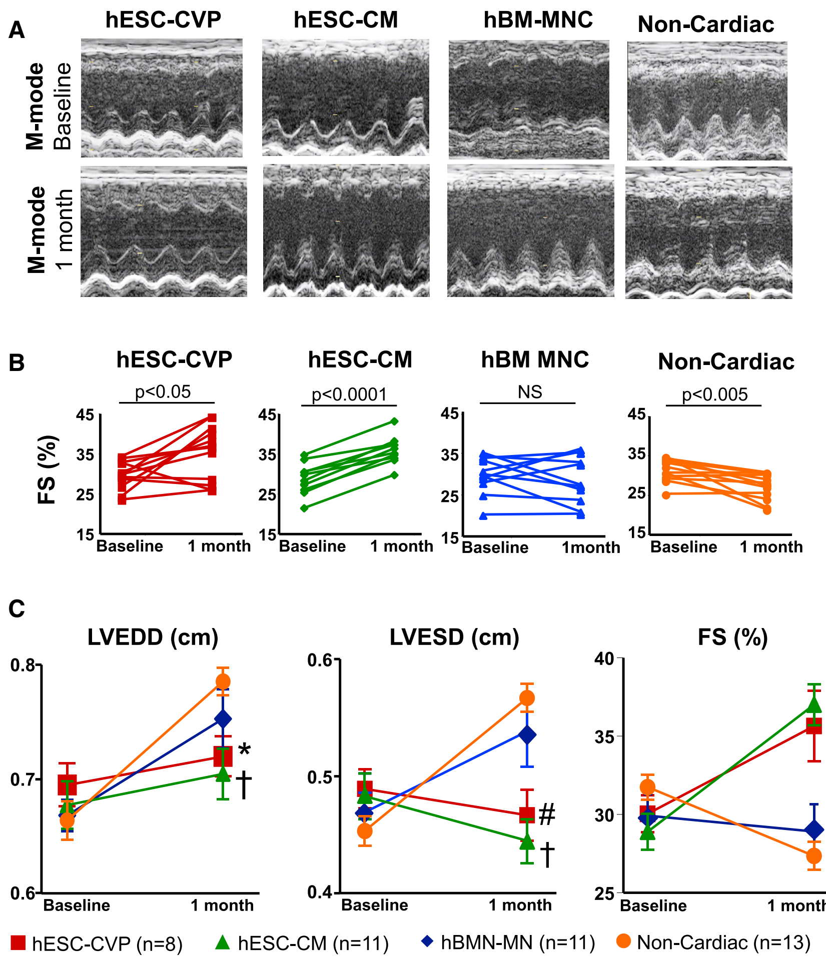

*, **, # and † represent p< 0.005, p<0.001, p<0.0005 and p<0.0001 against Non Cardiac group respectively

Figure 2. Cardiac Function after Cell Injection

Evaluation was performed showing the effects of cell transplantation on post-infarct ventricular function by echocardiography at 28 days after cell injection.

(A) Representative short axis of M-mode images of hearts of hESC-CVP, hESC-CM, BM-MNC, and non-cardiac recipients at baseline and at the 1-month time point. There was reduced ventricular dilation and increased contractility in the hESC-CM and hESC-CVP recipients compared with non-cardiac or BM-MNC recipients. Scale bar represents 1 cm.

(B) Individual assessments of the infarcted rats before (baseline) and 1 month after cell injection show a global increase of fractional shortening (FS) in hESC-CVP- and hES-CM-injected animals. Fractional shortening remained steady in BM-MNC-injected hearts and significantly decreased in the non-cardiac recipients.

(C) Transplantation of hESC-CMs or hESC-CVPs was associated with a decrease in LVEDD and a steady LVESD, resulting in a significant increase in fractional shortening at 4 weeks. In contrast, LVEDD and LVESD increased in both BM-MNC and non-cardiac recipients, and fractional shortening decreased significantly in the non-cardiac recipients (hESC-CVP, n = 8; hESC-CM, n = 11; BM-MNC, n = 11; and non-cardiac recipients, n = 13).

the different models, cell processing procedures, delivery, and varying endpoints, comparison of different cell sources is difficult. In fact, direct comparison has been addressed in only a few studies. When transplanted in a rabbit infarct model, both dermal fibroblast and skeletal myoblasts improved cardiac compliance, but only myoblasts improved systolic function (Hutcheson et al., 2000). In a rat model, both skeletal myoblasts and fetal cardiomyocytes showed heart function improvement to the same extent (Scorsin et al., 2000). In three other studies, cardiomyocytes and skeletal myoblast were both compared with marrow-derived cells. In both cases, their transplantation in a myocardial injury model improved cardiac function to a similar degree as bone marrow-derived cells (Paulis et al., 2013; Thompson et al., 2003; Yau et al., 2003). Although these studies indicate that implanting multiple cell types improves function, not every cell type is suitable for clinical use.

Our study compared human BM-MNCs (the cell type has been widely used in clinical trials) with two hESC derivatives. This cardiac cell therapy was studied to compare the efficacy of two cardiovascular derivatives of hESCs with a cell type currently in clinical trials (human BM-MNCs). By comparing to control non-cardiac derivatives of hESCs (a cell source proven to have no effect on cardiac function after transplantation) (Laflamme et al., 2007; Shiba et al., 2012; van Laake et al., 2007), we confirmed that hBM-MNCs do have a modest but significant beneficial effect on cardiac contractile function. As was previously showed in preclinical studies, we did not observe any cell survival 1 month after the injection of BM-MNCs (Kocher et al., 2001; Paulis et al., 2013; van der Bogt et al., 2008). Taken together, those results prove that the preservation of cardiac function by hBM-MNC transplantation is not attributable to transdifferentiation of the injected cells into cardiomyocytes. Rather, benefit appears to be attributable to an augmentation of vascularization in the area remote from the infarct, confirming the paracrine hypothesis of such therapeutic strategy. Interestingly, using tracer microspheres to measure myocardial blood flow, we

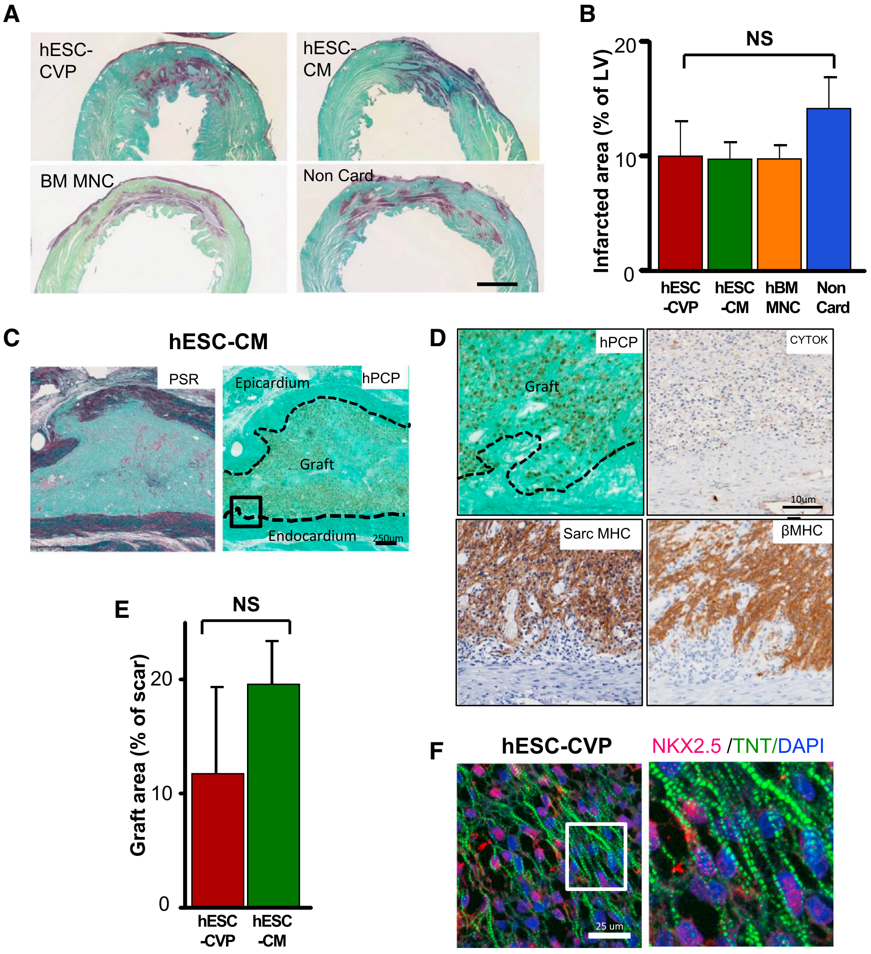

Figure 3. Histological Evaluation of Human Grafts at 1 Month after Transplantation

(A) Representative picrosirius red/fast green-stained histological sections from recipients of hESC-CVP, hESC-CM, hBM-MNC, and non-cardiac hESC derivatives at day 28 after cell transplantation. Note that all animals showed the presence of scar tissue (red) within the left ventricular segment. Scale bar represents 2 mm.

(B) Infarct size expressed as a percentage of left ventricular area was not significantly different among the four groups (hESC-CVP, n = 4; hESC-CM, n = 10; hBM-MNC, n = 10; and non-cardiac hESC-derivatives, n = 4).

(C) Serial sections of a hESC-CM graft stained with fast green/picrosirius red (left) and detected by in situ hybridization using hPCP (right).

(D) Detail of region within black box from (C) showing the grafts contained numerous human cells (hPCP), rich in cardiomyocytes (sarcomeric MHC and βMHC), and devoid of epithelial elements (cytokeratin, CYTOK).

(E) Human graft size was not significantly different between the hESC-CVP and hESC-CM recipients (n = 4 and n = 10, respectively).

(F) Confocal imaging of the human graft (human NKX2.5 pink), demonstrating presence of contractile apparatus (Troponin T; green).

did not observe any significant difference in blood flow among any of our groups. One explanation could be that an angiogenic response is occurring at the post-capillary level, downstream of the main resistance vessels that determine overall blood flow (Figure S2C). This would imply that blood flow in these neovessels is limited compared with the uninjured circulation and may not provide substantial nutrient value. Interventions that induce hierarchical remodeling of the upstream vessels (arteriogenesis) would be expected to have greater impact on flow than those that only grow new capillaries. Although in our study injection of hESC-CVPs or hESC-CMs did not increase cardiac vascularization when compared with our non-cardiac cell control group, we cannot infer a total absence of angiogenic effect, since injection of non-cardiac derivatives could possibly favor angiogenesis. Inclusion of a vehicle only or no injection control group would be necessary to confirm the absence of neovascularization.

One hypothesis leading into this study was that hESC-CVPs would be superior to hESC-CMs by giving larger, more widely distributed grafts that contained human cardiomyocytes and human vessels. This hypothesis was not supported by the data. Instead, the hESC-CVP grafts tended to be smaller than the hESC-CM grafts, and neither group showed significant numbers of human vessels. It is possible that the hESC-CVP group could be further improved, e.g., by co-delivering factors that promote endothelial differentiation, expansion, and survival, but it seems clear that host factors alone do not support infarct vascularization from these cells.

An interesting finding of our present study is that hESC-CMs not only blocked adverse dilation, as previously observed, but also led to improved fractional shortening. This improvement in contractility was not observed in our previous studies with the same animal model (Laflamme et al., 2007). One factor that could explain the additional beneficial effect is use of a different cell differentiation protocol, leading to a cell therapy product that contains not only cardiomyocytes but also endothelial cells and smooth muscle cells (whereas in the previous cell differentiation protocol, smooth muscle and endothelial cells were absent from the final product; Laflamme et al., 2007;

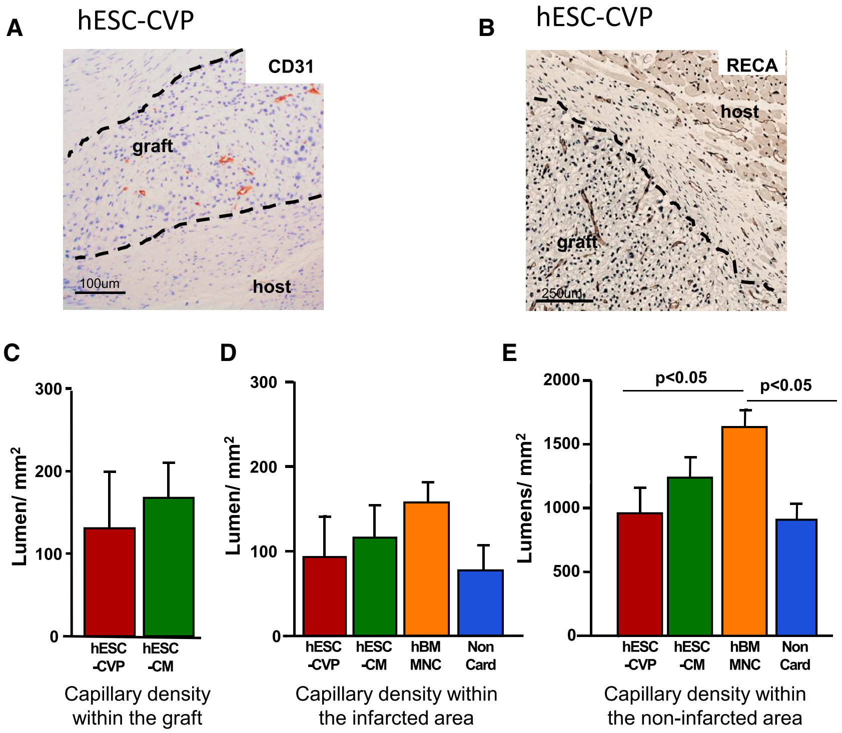

Figure 4. Evaluation of Revascularization at 1 Month after Cell Injection

(A) Human specific CD31 immunostaining showing rare human-derived vessels within the graft.

(B) Rat endothelial cell (RECA) immunostaining of heart injected with hESC-CVPs showing capillary density within the graft is sparse when compared with the adjacent host tissue.

(C) Vascularization of the human graft is similar in hESC-CM and hESC-CVP recipients (as human grafts were absent from the non-cardiac and hBM-MNC groups, graft vascularization could not be evaluated in those groups).

(D and E) Scar vascularization did not differ between groups (D). However, in the area remote from the scar zones (E), we observed enhanced vascularization in the hBM-MNC group.

(C–E) hESC-CVP, n = 4; hESC-CM, n = 10; hBM-MNC, n = 10; and non-cardiac hESC derivatives, n = 4.

Fernandes et al., 2010). This suggests that cell types other than cardiomyocytes are essential in the beneficial effect of cardiac cell therapy. This beneficial effect can be mediated by the secretion of paracrine factors or by facilitating the delivery of paracrine factor through neoangiogenesis. Another potential difference is the use of a modified pro-survival cocktail in the current study. In any case, FS of the hESC-CVP and hESC-CM group reach only 35% and 37%, significantly below that of the normal nude rat (51% ± 2%; n = 9, data not shown), indicating that the function was not completely restored and leaving room for further improvement.

A major finding of our study is that both hESC-CVPs and hESC-CMs showed a superior beneficial effect on cardiac function when compared with hBM-MNCs. This study also suggests distinct mechanisms of action between the hESC-derived cardiomyocytes and hBM-MNC cell delivery: remuscularization, enhanced vascularization of the host myocardium, or release of paracrine factors. Our group recently used guinea pig and macaque cardiac injury models to show that, once injected into the heart, hESC-CMs can couple and beat in synchrony with the host myocardium (Chong et al., 2014; Shiba et al., 2012) at rates up to 240 beat per minute. However, we have not measured electromechanical coupling in this study. Therefore, we cannot comment on the extent to which the observed benefit results from new force generating units versus paracrine effects. There was somewhat greater mortality in the hESC-CM group (27%) compared with the hBM-MNC group (9%), and we cannot exclude earlier deaths of animals with larger infarcts. Since after excluding these animals both groups had comparable pre-treatment cardiac dysfunction, this difference is unlikely to influence the treatment effect.

Although we attempted to reproduce key clinical elements such as the use of human cell products and an ischemia-reperfusion model in the host, a few caveats should be emphasized in applying these data to humans. Importantly, our animals did not receive any additional pharmacological treatment such as beta-adrenergic receptor blockers or angiotensin-converting enzyme inhibitors. Interestingly, preclinical studies performed with skeletal myoblasts demonstrated that the benefits of cell transplantation lasted longer than the benefit associated with angiotensin-converting enzyme inhibition (Fujii et al., 2003) and that cells and pharmacological beneficial effects were additives (Pouzet et al., 2001). This demonstrated that cell transplantation can augment cardiac function in the context of standard pharmacological treatment. Another limitation pertains to the use of a xenotransplantation model, which precludes learning how adaptive immune responses would evolve in the setting of allogenic cell therapy in humans. Importantly, our preclinical study assessed the change in cardiac function only within the first 28 days after cell transplantation. It is unknown whether the cardiac functional improvement will be sustained at later time points. It is noteworthy that hESC-CM delivery in a

murine model of myocardial infarction similarly demonstrated cardiac functional improvements at 1 month; however, this was not sustained on reassessment at 3 months (van Laake et al., 2007; van Laake et al., 2008). Further studies would need to be performed to evaluate the long-term beneficial effect of the hESC-CM protocol used in the current study. Finally, cells were delivered by direct injection into the infarct region, whereas in clinical trials, the predominant route of BM-MNC delivery is by intra-coronary injection.

In summary, using direct side-by-side comparison, associated with a blinded analysis of cardiac function, we demonstrated the superiority of hESC cardiac derivatives over BM-MNC transplantation in improving cardiac function 28 days after transplantation. These data, combined with our recent report demonstrating that hESC-CM transplantation remuscularizes a substantial fraction of the non-human primate infarct with synchronously beating human myocardium (Chong et al., 2014), support continued research in the use of cardiomyocytes derived from pluripotent human stem cells for heart regeneration.

EXPERIMENTAL PROCEDURES

Preparation of hESC-Derived Cardiomyocytes

To ensure comparability, we derived the cardiomyocytes, progenitors, and non-cardiac derivative used for this study from the same batch of human ESC (H7) that was expanded and cryopreserved until thawing for differentiation.

The expansion phase of undifferentiated hESCs was performed on mouse embryonic fibroblasts (MEFs) in medium consisting of DMEM/F12 supplemented with 20% KnockOut serum replacement (Invitrogen), L-glutamine, non-essential amino acids, beta-mercaptoethanol, and BFGF (10 ng/ml; Peprotech). Cells were passaged using collagenase IV and trypsin, as well as the ROCK inhibitor Y-27632 (Rho-associated kinase inhibitor; 10 μM, Tocris) to enhance cell survival. Human ESCs were depleted of MEFs by at least two passages on Matrigel-coated plates before cryopreservation. For growth on Matrigel, cells were maintained in MEF-conditioned medium (MEF-CM) supplemented with 8-ng/ml BFGF.

Cardiac differentiation was induced using an embryoid body method previously described (Yang et al., 2008). For embryoid body formation, cells were resuspended into low-attachment plates in StemPro-34 medium (Invitrogen) supplemented with L-glutamine (15 mg/ml), ascorbic acid (50 μg/ml), transferrin (150 μg/ml), and monothioglycerol (50 μg/ml). For further direction of cell differentiation toward the cardiovascular lineage, the basal media was supplemented with 0.5 ng/ml bone morphogenic protein 4 (BMP4, R&D) for 24 hr followed by 10 ng/ml BMP4, 6 ng/ml Activin A (R+D), and 5 ng/ml BFGF for 3 days. On day 4 of differentiation, the embryoid bodies were dissociated into single cells using trypsin and seeded onto Matrigel-coated plates at a density of 10^5 cells/cm^2 in StemPro. Medium was changed every 3–4 days thereafter until day 14 of differentiation. For non-cardiac preparations (principally neuroectoderm), we used the same batch of hESCs (H7), but differentiation was performed under monolayer condition, as previously described (Fernandes et al., 2010; Laflamme et al., 2007).

Cell Preparation before Injection

At 1 day prior injection, hESC derivatives (progenitors and definitive and non-cardiac derivatives) were subjected to a pro-survival protocol, modified from that previously shown to enhance engraftment post-transplantation (Fernandes et al., 2010; Laflamme et al., 2007). In brief, at days 4 or 14 of differentiation for hESC-CVPs or hESC-CMs, respectively, cultures were heat shocked with a 30-min exposure to 43°C media, followed by a return at 37°C in fresh media supplemented with cyclosporine A (to close the mitochondrial transition pore; 0.2 μM, Sandimmune, Novartis). One day later, cultures underwent a 1-hr pretreatment with Y-27632 (Rho-associated kinase inhibitor; 10 μM) and then were harvested with 0.25% trypsin/0.5 mM EDTA (Invitrogen). After being washed with DMEM/F12 supplemented with DNase (Invitrogen, 100 U/ml), 10 × 10^6 cells were suspended in a 100 μl-volume per animal. The injection vehicle consisted of growth factor-reduced Matrigel in basal StemPro-34 medium (50% vol/vol), supplemented with L-glutamine, ascorbic acid, transferrin, monothioglycerol, cyclosporine A (200 nM, Wako), and Y-27632 (10 μM).

Fresh bone marrow mononuclear cells were purchased from AllCells LLC (ABM024). After overnight shipment, they were washed twice in DMEM/F12 and resuspended at the same density and in the same vehicle as hESC derivatives (10 × 10^6 cells per 100 μl).

For immunophenotyping purpose, aliquots of each cell batch were preserved for flow cytometry analysis at the time of cell injection.

Myocardial Infarction Model and Cell Transplantation

All animal experiments were performed in accordance with the Guide for the Care and Use of Laboratory Animals published by the U.S. NIH (NIH Publication No. 85-23, revised 1996) and were approved by our institutional animal care and use committee. The myocardial infarction model and cell transplantation protocol have been described previously in several reports by our group (Fernandes et al., 2010; Laflamme et al., 2007). In brief, myocardial infarction of athymic male Sprague Dawley rats (rnu-rnu, 200–250 g, Charles Rivers) was induced by 60 min of I/R injury (ligation of the left anterior descending artery by 7-0 Prolene suture). Four days after I/R, animals underwent echocardiographic evaluation, and animals with fractional shortening >40% were excluded on the basis of small infarcts. Qualified animals were randomly assigned to one of the four treatment groups (Figure 1). A total of 10 × 10^6 cells were injected at three sites within the left ventricular wall (two injections at the lateral borders of the infarct and one in the central ischemic zone). All rats received daily subcutaneous injections of cyclosporine A (0.75 mg/day, Wako Pure Chemicals a sub-immunosuppressive dose that should facilitate mitochondrial permeability pore closure) starting 1 day before engraftment and continuing for 7 days after engraftment.

Flow Cytometry

Human ESC-CMs were analyzed by flow cytometry at the cardiovascular progenitor stage, definitive stages (days 4 and 14 of

differentiation, respectively). For non-cardiac derivative and bone marrow mononuclear cells, a sample of the cells was kept at the time of injection. Primary antibodies are detailed in Table S1, and the flow cytometry analysis was performed using a BD FACS Canto. Gating parameters were defined using cells incubated with only the secondary antibody and omitting the primary antibody.

Cardiac Function Evaluation

Cardiac function was evaluated by echocardiography on lightly anesthetized animals (2% isoflurane; Novaplus). Left ventricular end-diastolic dimension (LVEDD), end-systolic dimension (LVESD), and heart rate were measured by transthoracic echocardiography (GE Vivid 7) with a 10S (10 MHz) pediatric transducer. Fractional shortening was calculated by this equation: FS = 100 × (LVEDD – LVESD)/LVEDD. All cardiac function evaluations (echocardiography and their analysis) were performed by an investigator blinded to the respective treatment.

Neutron-Activated Microspheres and Tissue Processing

Evolution of cardiac blood flow after cell injection was evaluated using the neutron-activated microsphere technique (Reinhardt et al., 2001). Gold and Samarium microspheres (200,000 of 15-μm diameter in 200 μl; BioPAL) were injected into the left ventricular chamber via a 25G catheter at a controlled rate of 300 ml/hr at the time of cell injection and at the time of sacrifice, respectively. Microspheres were allowed to circulate for 5 min, and chest was either closed (at the 4-day time point) or heart was arrested by injection of potassium chloride (at the 1-month time point). At the time of sacrifice, hearts were harvested, perfused with PBS, and then paraformaldehyde (4%, 50 ml, 80–100 mmHg pressure). Hearts were cut into short-axis sections using a rat heart slicer matrix (Zivic Instruments). Four sections per heart were collected starting at the apex (Figure S2); the remaining heart tissue (closer to the base) was discarded. At least two sections per heart were at stored at 4°C until further processing for histological analysis. The two other sections were used for microsphere analysis, and the infarct and uninjured segment of each section were isolated under visualization of a dissecting microscope. Infarct and non-infarcted tissues were weighed and were sent for analysis (BioPAL). For calculation of relative regional myocardial blood flow, relative decay per minute of the pooled infarct samples and uninjured samples was normalized to tissue weight. Blood flow to the infarct region was expressed as a percentage of that in the non-infarcted region using the following formula: 100 × (relative decay per minute in infarct region / tissue weight) / (relative decay per minute in uninjured region / tissue weight).

Histology

Histological studies were performed as previously detailed by our group (Fernandes et al., 2010; Laflamme et al., 2007). For immunohistochemistry, we used the primary antibodies detailed in Table S2, followed by either fluorescent secondary antibodies (Alexa-conjugated, species-specific antibodies from Molecular Probes) or the avidin-biotin reaction followed by chromogenic detection (ABC kits from Vector Labs). In situ hybridization against the human-specific pan-centromeric probe was performed using methods previously detailed (Laflamme et al., 2007). For detection, we used a peroxidase-conjugated anti-digoxigenin antibody (Roche), followed by staining with either a chromogenic substrate or fluorescent tyramide signal amplification kit (Molecular Probes).

Statistical Analysis

All values were expressed as mean ± SEM. Statistical analyses were performed using Graphpad 4.0 with the threshold for significance set at level $p < 0.05$. Echocardiographic outcomes were analyzed by ANOVA followed by post hoc comparisons between groups by Tukey HSD. For infarct size and left ventricular thickness, groups were compared using a one-way ANOVA followed by a Kruskal Wallis test. A Student's t test was used to compare graft size between hESC-CMs and CVPs.

SUPPLEMENTAL INFORMATION

Supplemental Information includes three figures and four tables and can be found with this article online at http://dx.doi.org/10.1016/j.stemcr.2015.09.011.

AUTHOR CONTRIBUTIONS

S.F. designed and performed cell and animal experiments, analyzed data, and wrote the manuscript. J.J.H.C. designed and performed cell and animal experiments, analyzed data, and wrote the manuscript. M.I. performed cell experiments and analyzed data. B.T.-S. oversaw bone marrow experiments and analyzed data. G.K. oversaw hESC experiments and analyzed data. H.R. designed cell and animal experiments, analyzed data, and contributed to manuscript writing, and C.E.M. obtained research funding, designed cell and animal experiments, analyzed data, oversaw entire project, and contributed to manuscript writing.

ACKNOWLEDGMENTS

We thank Sarah Dupras, Mark Saiget, Nina Tan, Luz Linarez, and Veronica Muskeli for their technical assistance. This work was supported by NIH grants P01 HL094374, R01 HL084642, P01 GM081619, and U01 HL100405. S.L.P. was supported through NIH F30 HL095343. J.J.H.C. was supported by National Health and Medical Research Council of Australia Overseas Training and Australian-American Fulbright Commission Fellowships.

Received: November 14, 2013
Revised: September 14, 2015
Accepted: September 15, 2015
Published: October 22, 2015

REFERENCES

Caspi, O., Huber, I., Kehat, I., Habib, M., Arbel, G., Gepstein, A., Yankelson, L., Aronson, D., Beyar, R., and Gepstein, L. (2007). Transplantation of human embryonic stem cell-derived cardiomyocytes improves myocardial performance in infarcted rat hearts. J. Am. Coll. Cardiol. *50*, 1884–1893.

Chong, J.J. (2012). Cell therapy for left ventricular dysfunction: an overview for cardiac clinicians. Heart Lung Circ. *21*, 532–542.

Chong, J.J., Yang, X., Don, C.W., Minami, E., Liu, Y.W., Weyers, J.J., Mahoney, W.M., Van Biber, B., Cook, S.M., Palpant, N.J., et al. (2014). Human embryonic-stem-cell-derived cardiomyocytes regenerate non-human primate hearts. Nature *510*, 273–277.

Dai, W., Kay, G.L., Jyrala, A.J., and Kloner, R.A. (2013). Experience from experimental cell transplantation therapy of myocardial infarction: what have we learned? Cell Transplant. *22*, 563–568.

Fernandes, S., Naumova, A.V., Zhu, W.Z., Laflamme, M.A., Gold, J., and Murry, C.E. (2010). Human embryonic stem cell-derived cardiomyocytes engraft but do not alter cardiac remodeling after chronic infarction in rats. J. Mol. Cell. Cardiol. *49*, 941–949.

Fujii, T., Yau, T.M., Weisel, R.D., Ohno, N., Mickle, D.A., Shiono, N., Ozawa, T., Matsubayashi, K., and Li, R.K. (2003). Cell transplantation to prevent heart failure: a comparison of cell types. Ann. Thorac. Surg. *76*, 2062–2070, discussion 2070.

Hansson, E.M., Lindsay, M.E., and Chien, K.R. (2009). Regeneration next: toward heart stem cell therapeutics. Cell Stem Cell *5*, 364–377.

Hutcheson, K.A., Atkins, B.Z., Hueman, M.T., Hopkins, M.B., Glower, D.D., and Taylor, D.A. (2000). Comparison of benefits on myocardial performance of cellular cardiomyoplasty with skeletal myoblasts and fibroblasts. Cell Transplant. *9*, 359–368.

Kocher, A.A., Schuster, M.D., Szabolcs, M.J., Takuma, S., Burkhoff, D., Wang, J., Homma, S., Edwards, N.M., and Itescu, S. (2001). Neovascularization of ischemic myocardium by human bone-marrow-derived angioblasts prevents cardiomyocyte apoptosis, reduces remodeling and improves cardiac function. Nat. Med. *7*, 430–436.

Laflamme, M.A., Chen, K.Y., Naumova, A.V., Muskheli, V., Fugate, J.A., Dupras, S.K., Reinecke, H., Xu, C., Hassanipour, M., Police, S., et al. (2007). Cardiomyocytes derived from human embryonic stem cells in pro-survival factors enhance function of infarcted rat hearts. Nat. Biotechnol. *25*, 1015–1024.

Leor, J., Gerecht, S., Cohen, S., Miller, L., Holbova, R., Ziskind, A., Shachar, M., Feinberg, M.S., Guetta, E., and Itskovitz-Eldor, J. (2007). Human embryonic stem cell transplantation to repair the infarcted myocardium. Heart *93*, 1278–1284.

Loffredo, F.S., Steinhauser, M.L., Gannon, J., and Lee, R.T. (2011). Bone marrow-derived cell therapy stimulates endogenous cardiomyocyte progenitors and promotes cardiac repair. Cell Stem Cell *8*, 389–398.

Paulis, L.E., Klein, A.M., Ghanem, A., Geelen, T., Coolen, B.F., Breitbach, M., Zimmermann, K., Nicolay, K., Fleischmann, B.K., Roell, W., and Strijkers, G.J. (2013). Embryonic cardiomyocyte, but not autologous stem cell transplantation, restricts infarct expansion, enhances ventricular function, and improves long-term survival. PLoS ONE *8*, e61510.

Pouzet, B., Ghostine, S., Vilquin, J.T., Garcin, I., Scorsin, M., Hagège, A.A., Duboc, D., Schwartz, K., and Menasché, P. (2001). Is skeletal myoblast transplantation clinically relevant in the era of angiotensin-converting enzyme inhibitors? Circulation *104* (*Suppl 1*), I223–I228.

Reinhardt, C.P., Dalhberg, S., Tries, M.A., Marcel, R., and Leppo, J.A. (2001). Stable labeled microspheres to measure perfusion: validation of a neutron activation assay technique. Am. J. Physiol. Heart Circ. Physiol. *280*, H108–H116.

Scorsin, M., Hagège, A., Vilquin, J.T., Fiszman, M., Marotte, F., Samuel, J.L., Rappaport, L., Schwartz, K., and Menasché, P. (2000). Comparison of the effects of fetal cardiomyocyte and skeletal myoblast transplantation on postinfarction left ventricular function. J. Thorac. Cardiovasc. Surg. *119*, 1169–1175.

Shiba, Y., Fernandes, S., Zhu, W.Z., Filice, D., Muskheli, V., Kim, J., Palpant, N.J., Gantz, J., Moyes, K.W., Reinecke, H., et al. (2012). Human ES-cell-derived cardiomyocytes electrically couple and suppress arrhythmias in injured hearts. Nature *489*, 322–325.

Thompson, R.B., Emani, S.M., Davis, B.H., van den Bos, E.J., Morimoto, Y., Craig, D., Glower, D., and Taylor, D.A. (2003). Comparison of intracardiac cell transplantation: autologous skeletal myoblasts versus bone marrow cells. Circulation *108* (*Suppl 1*), II264–II271.

van der Bogt, K.E., Sheikh, A.Y., Schrepfer, S., Hoyt, G., Cao, F., Ransohoff, K.J., Swijnenburg, R.J., Pearl, J., Lee, A., Fischbein, M., et al. (2008). Comparison of different adult stem cell types for treatment of myocardial ischemia. Circulation Suppl. *118*, S121–S129.

van Laake, L.W., Passier, R., Monshouwer-Kloots, J., Nederhoff, M.G., Ward-van Oostwaard, D., Field, L.J., van Echteld, C.J., Doevendans, P.A., and Mummery, C.L. (2007). Monitoring of cell therapy and assessment of cardiac function using magnetic resonance imaging in a mouse model of myocardial infarction. Nat. Protoc. *2*, 2551–2567.

van Laake, L.W., Passier, R., Doevendans, P.A., and Mummery, C.L. (2008). Human embryonic stem cell-derived cardiomyocytes and cardiac repair in rodents. Circ. Res. *102*, 1008–1010.

Yang, L., Soonpaa, M.H., Adler, E.D., Roepke, T.K., Kattman, S.J., Kennedy, M., Henckaerts, E., Bonham, K., Abbott, G.W., Linden, R.M., et al. (2008). Human cardiovascular progenitor cells develop from a KDR+ embryonic-stem-cell-derived population. Nature *453*, 524–528.

Yau, T.M., Tomita, S., Weisel, R.D., Jia, Z.Q., Tumiati, L.C., Mickle, D.A., and Li, R.K. (2003). Beneficial effect of autologous cell transplantation on infarcted heart function: comparison between bone marrow stromal cells and heart cells. Ann Thorac Surg *75*, 169–176, discussion 176–167.

Stem Cell Reports
Supplemental Information

Comparison of Human Embryonic Stem Cell-Derived Cardiomyocytes, Cardiovascular Progenitors, and Bone Marrow Mononuclear Cells for Cardiac Repair

Sarah Fernandes, James J.H. Chong, Sharon L. Paige, Mineo Iwata, Beverly Torok-Storb, Gordon Keller, Hans Reinecke, and Charles E. Murry

Supplementary Data

Supplementary Figures Legend

Supplementary Figure S1. Human ESC differentiation protocols. Human ESC derivatives were obtained from a unique batch of cryopreserved undifferentiated ESC. Human ESCs were depleted of MEFs by at least 2 passages on Matrigel coated plate, before cryopreservation. Non-cardiac derivatives were obtained using a monolayer differentiation protocol. Progenitor and definitive cells were issued from the same differentiation protocol: Cardiovascular progenitors and definitive cells were obtained at day 5 and 15 after the beginning of the differentiation respectively. All cells underwent a heat shock procedure the day before being harvested for intracardiac injection. * Sampling for flow cytometry analysis.

Supplementary Figure S2. Evaluation of vascularization using neutron activated microsphere. a Sample preparation**:** After overnight fixation, hearts were sliced in 5 sections. The basal section was discarded, 2 sections were kept for histology and 2 for microsphere analysis (flow). For blood flow assessment infarcted and uninjured segment were individually isolated, whereas peri infarcted area were discarded. RV, right ventricle; LV, left ventricle**. b** Evaluation of the vascularization using microsphere injections in the non infarcted area did not show any significant difference between the 4 groups at one month time point. **c** Representative RECA staining of BM-MNC group showing that capillary staining and the presence of 2 microspheres (red arrows) within a non infarcted area.

Supplementary Figure S3. Immunophenotyping of human grafts in Non-Cardiac recipients. Photomicrograph of the largestt human graft observed in the Non-Cardiac recipients. Adjacent sections have been stained with Hematoxylin Eosin, human Pan Centromeric probe, beta Myosin Heavy Chain and, pan cytokeratin. Immunostainings confirmed that, in the Non cardiac recipient, human grafts were mostly composed by epithelial derivatives, and were depleted of cardiomyocytes.

Supplementary Table S1. Primary antibodies used for cell immunophenotyping before cell transplantation

Antigen	Antibody Type (Clone Name or Catalog #)	Supplier	Titer
Cardiac troponin T	Mouse monoclonal Clone 11-13 (cat # MS-295)	LabVision/ Neomarkers	1:10
CD117/cKit-APC	Clone 104D2 (cat # 313205)	BioLegend	1:5
CD133-PE	Clone 293 C3 (cat # 130-090-8530)	Miltenyi Biotech	1:5
CD13-APC	Clone WM15 (cat # 5574540)	BD Biosciences	1:5
CD146-FITC	Clone P1H12 (cat # 5608460)	BD Biosciences	1:5
CD14-PE	Clone MφP3 (cat # 347497)	BD Biosciences	1:5
CD19-FITC	Clone HIB19 (cat # 560994)	BD Biosciences	1:5
CD235a/Glycopholin A-APC	Clone GA-R2(HIR2) (cat # 551336)	BD Biosciences	1:5
CD33-FITC	Clone HIM3-4 (cat # 561818)	BD Biosciences	1:5
CD34-APC	Clone 581 (cat # 555824)	BD Biosciences	1:5
CD3-PE	Clone SK7 (cat # 347347)	BD Biosciences	1:5
CD41a-APC	Clone HIP8 (cat # 559777)	BD Biosciences	1:5
CD45-PE	Clone HI30 (cat # 555484)	BD Biosciences	1:5
CD4-APC	Clone 11830 (cat # FAB3791A)	R&D Systems	1:5
CD8-APC	Clone 37006 (cat # FAB1509A)	R&D Systems	1:5
CD90/THY1-FITC	Clone F15-42-1 (cat # a b11155)	abcam	1:5
human CD31-PerCP-eFlour710	Mouse monoclonal Clone WM-59 (cat # 46-0319-41)	eBioscience	1:10
PDGFRα-APC	Mouse monoclonal Clone PRa292 (cat # FAB1264)	R & D Systems	1:5
smooth muscle actin	Rabbit polyclonal (cat # ab5694)	Abcam	1:5
VEGFR2/KDR-PE	Mouse monoclonal Clone 89106 (cat # FAB357)	R & D Systems	1:5

Supplementary Table S2. Primary antibodies used for histology analysis.

Antigen	Antibody Type (Clone Name or Catalog #)	Supplier	Titer
**Cadherin**	Mouse monoclonal Clone CH19	Sigma-Aldrich	1:100
**Cardiac troponin T**	Mouse monoclonal Clone 11-13 (cat # MS-295)	LabVision/ Neomarkers	1:400
**CD31 (Human-specific)**	Mouse monoclonal (Clone JC70A)	Dako	1:20
**Nkx2.5**	Horse polyclonal (cat.# AF2444)	R&D Systems	1/400
**Pan-cytokeratins**	Mouse monoclonal Clone AE1/AE3	Dako	1:150
**Reca**	Mouse monoclonal RECA- 1 (cat # ab9774)	abcam	1/15
**β3-tubulin**	Mouse monoclonal Clone SDL.3D10	Sigma-Aldrich	1:2000
**β-myosin heavy chain**	Mouse monoclonal Clone A4.951	ATCC	1:10

Abbreviations: ATCC— American Type Culture Collection

Supplementary Table S3. Characterization of BM-MNC preparations.

Antibody	%
CD117/cKit+	5±1
CD133+	<0.1
CD14+	10±1
CD146+	<0.1
CD19+	11±3
CD3+	24±3
CD33+, CD13+	23±3
CD34+	4±1
CD34+/cKit+	2±1
CD4+	12±2
CD41a+	3±1
CD45+	73±4
CD8+	11±2
CD90/Thy1+	<0.5
Glycophorin A+	11±2

Supplementary Table S4: Histomorphometry values of scar size evaluation

Group	Areas obtained by histomorphometry (arbitrary unit)									
	Section 1		Section 2		Section 3		Section 4			Scar/lv %
	LV	Scar	LV	Scar	LV	Scar	LV	Scar		
hESC-CVP	36.9	0.0	42.6	2.4	24.7	5.7	44.2	5.2		10.1
	27.2	0.0	40.4	11.5	20.4	7.3	41.4	4.0		18.5
	42.6	0.0	41.3	4.4	16.9	0.4				4.4
	63.8	5.0	30.6	3.9	48.5	0.0				6.9
									**Average**	**10.0**
hBM-MNC	32.4	5.5	21.8	4.4	30.8	1.0				13.5
	21.9	0.0	38.8	4.7	18.0	1.3	34.3	1.8		8.1
	29.9	0.0	36.3	4.9	30.1	0.0				4.5
	36.5	6.9	16.7	0.8	37.1	2.5				10.2
	45.4	8.8	18.2	4.6	44.4	0.0				14.9
	46.4	0.0	53.0	2.4	36.5	5.8	41.4	0.0		5.1
	34.8	0.0	49.9	4.3	45.8	10.7				10.6
	47.3	6.2	38.8	5.6	14.0	0.2				9.7
	38.0	0.0	45.3	2.0	39.8	4.8				5.5
	55.9	0.0	56.3	11.7	13.2	3.2				15.1
									**Average**	**9.7**
hESC-CM	33.5	1.5	36.9	6.6	21.8	2.7	36.9	2.5		12.3
	53.7	8.7	31.0	5.3	42.7	3.0				13.5
	38.5	5.2	22.1	2.4	38.8	1.8				9.6
	29.6	0.0	52.1	8.5	27.1	1.7	39.9	0.0		7.5
	59.6	0.0	66.7	13.4	28.6	5.1	68.3	0.0		12.6
	49.8	0.0	35.6	7.1	55.5	6.4	57.2	0.0		10.4
	33.7	0.0	47.7	2.2	23.6	0.3				1.9
									**Average**	**9.7**
Non cardiac	58.3	0.0	66.8	7.1	42.8	9.3				10.8
	70.0	0.0	72.8	8.7	35.5	17.1	83.9	11.0		18.3
	71.1	0.0	62.1	9.6	89.1	3.6	22.2	0.0		19.4
	55.1	0.0	49.6	6.9	27.8	2.9				8.1
									**Average**	**14.1**

Evaluation of scar was performed on picrosirius red stained section from base (section 1) to apex (section 3 or 4). Total left ventricular area (LV), scar area and scar/LV ratios were determined for each section. The scar/LV % was obtained by averaging the value of each section.

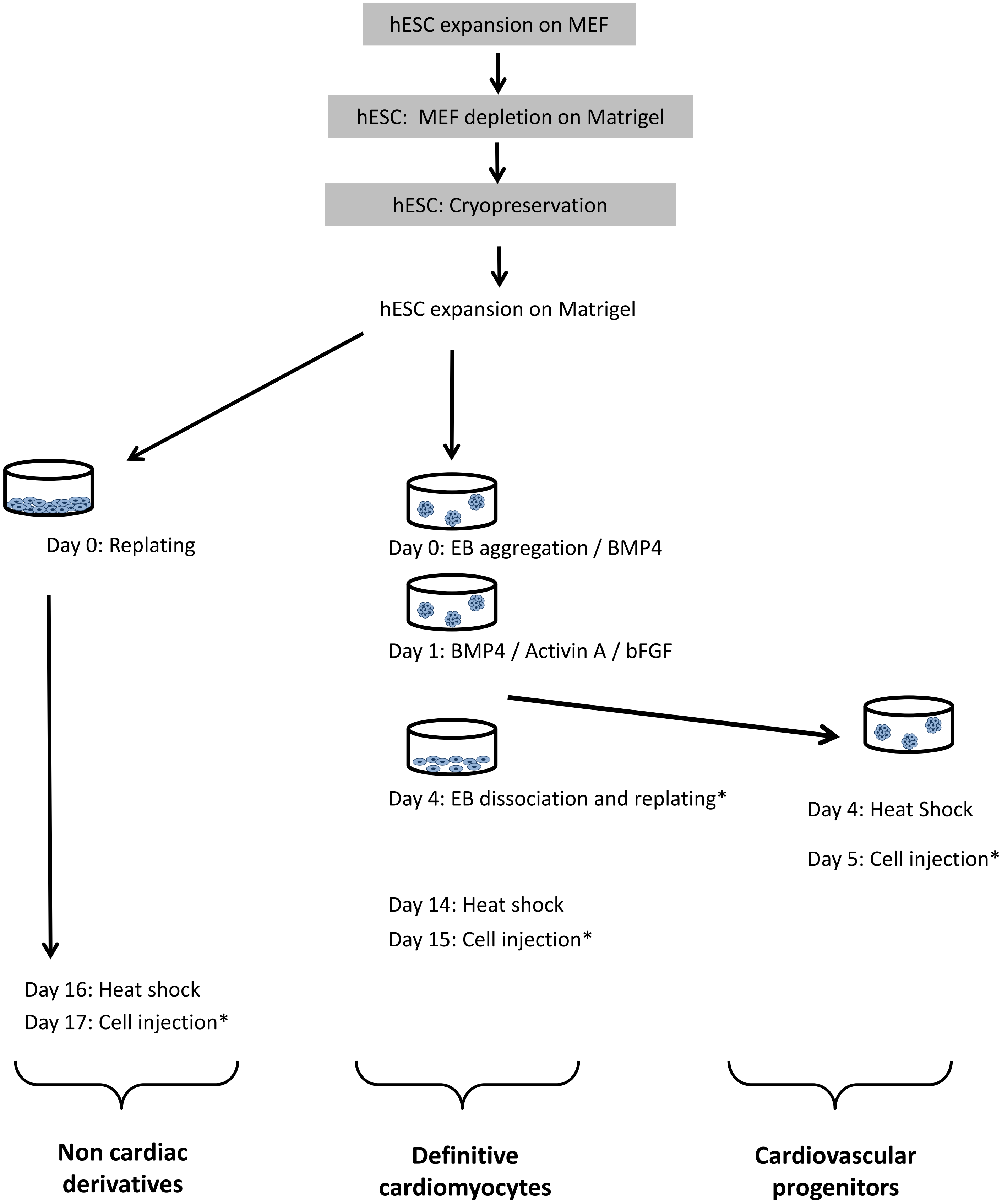

Supplementary figure S1

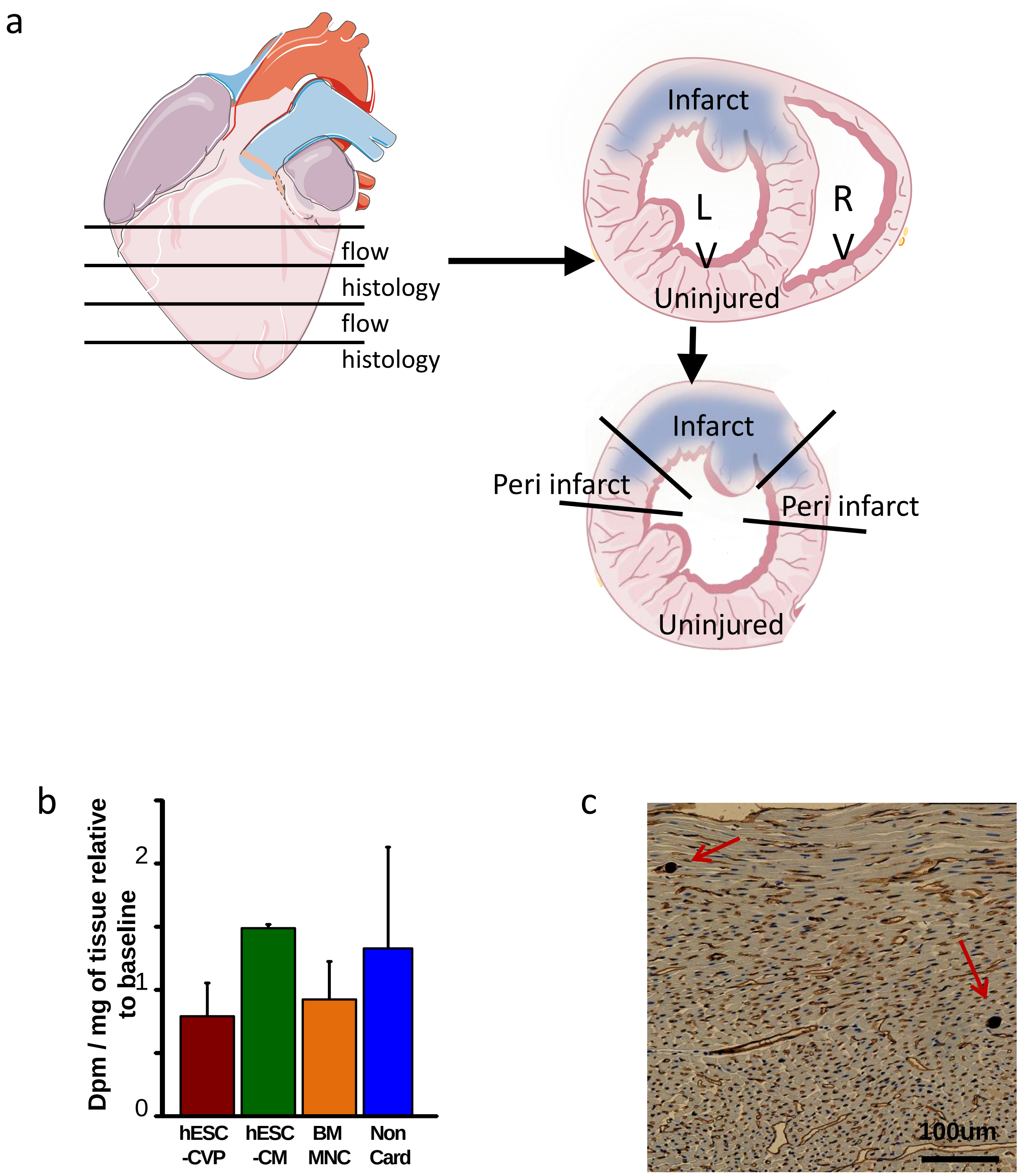

Supplementary Figure S2

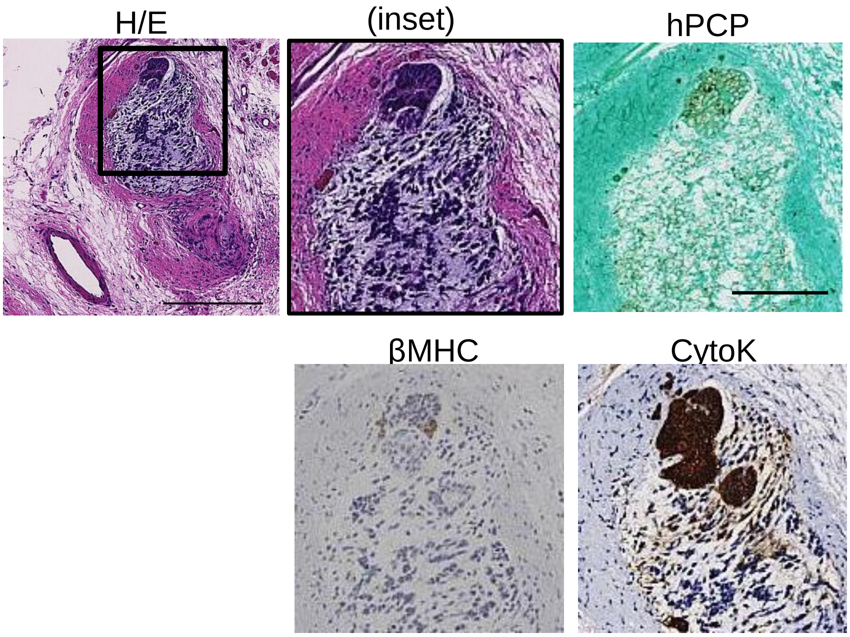

Supplementary Figure S3
